# Moving from frugivory to seed dispersal: Incorporating the functional outcomes of interactions in plant–frugivore networks

**DOI:** 10.1111/1365-2656.12831

**Published:** 2018-04-20

**Authors:** Benno I. Simmons, William J. Sutherland, Lynn V. Dicks, Jörg Albrecht, Nina Farwig, Daniel García, Pedro Jordano, Juan P. González‐Varo

**Affiliations:** ^1^ Conservation Science Group Department of Zoology University of Cambridge Cambridge UK; ^2^ Biological Sciences University of East Anglia Norwich UK; ^3^ Senckenberg Biodiversity and Climate Research Centre (BiK‐F) Frankfurt am Main Germany; ^4^ Conservation Ecology Faculty of Biology Philipps‐University Marburg Marburg Germany; ^5^ Departamento de Biología de Organismos y Sistemas Unidad Mixta de Investigación en Biodiversidad (CSIC‐UO‐PA) Universidad de Oviedo Oviedo Spain; ^6^ Integrative Ecology Group Estación Biológica de Doñana (EBD‐CSIC) Sevilla Spain

**Keywords:** antagonism, ecological networks, fleshy fruits, frugivorous birds, mutualism, mutualistic networks, pulp pecking, seed predation

## Abstract

1. There is growing interest in understanding the functional outcomes of species interactions in ecological networks. For many mutualistic networks, including pollination and seed dispersal networks, interactions are generally sampled by recording animal foraging visits to plants. However, these visits may not reflect actual pollination or seed dispersal events, despite these typically being the ecological processes of interest.
2. Frugivorous animals can act as seed dispersers, by swallowing entire fruits and dispersing their seeds, or as pulp peckers or seed predators, by pecking fruits to consume pieces of pulp or seeds. These processes have opposing consequences for plant reproductive success. Therefore, equating visitation with seed dispersal could lead to biased inferences about the ecology, evolution and conservation of seed dispersal mutualisms.
3. Here, we use natural history information on the functional outcomes of pairwise bird–plant interactions to examine changes in the structure of seven European plant–frugivore visitation networks after non‐mutualistic interactions (pulp pecking and seed predation) have been removed. Following existing knowledge of the contrasting structures of mutualistic and antagonistic networks, we hypothesized a number of changes following interaction removal, such as increased nestedness and lower specialization.
4. Non‐mutualistic interactions with pulp peckers and seed predators occurred in all seven networks, accounting for 21%–48% of all interactions and 6%–24% of total interaction frequency. When non‐mutualistic interactions were removed, there were significant increases in network‐level metrics such as connectance and nestedness, while robustness decreased. These changes were generally small, homogenous and driven by decreases in network size. Conversely, changes in species‐level metrics were more variable and sometimes large, with significant decreases in plant degree, interaction frequency, specialization and resilience to animal extinctions and significant increases in frugivore species strength.
5. Visitation data can overestimate the actual frequency of seed dispersal services in plant–frugivore networks. We show here that incorporating natural history information on the functions of species interactions can bring us closer to understanding the processes and functions operating in ecological communities. Our categorical approach lays the foundation for future work quantifying functional interaction outcomes along a mutualism–antagonism continuum, as documented in other frugivore faunas.

## Graphical abstract

There is growing interest in understanding functional roles in species interaction networks. Here, the authors use data on the functional outcomes of bird–plant interactions to examine changes in the structure of visitation networks after removing non‐mutualistic interactions. Incorporating this information brings us closer to understanding ecological processes of interest within networks. Photo by Saverio Gatto.

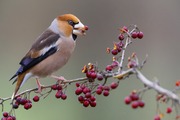

## Resumen

1. Existe un interés creciente en comprender las consecuencias funcionales de las interacciones entre especies en redes ecológicas. Para muchas redes mutualistas, incluidas las de polinización y dispersión de semillas, las interacciones se muestrean generalmente registrando las visitas de consumo que los animales realizan en las plantas. Sin embargo, tales visitas pueden no reflejar eventos reales de polinización o dispersión de semillas, a pesar de ser estos los procesos ecológicos de interés.
2. Los animales frugívoros pueden actuar como dispersores de semillas, si ingieren los frutos y dispersan sus semillas, o como consumidores de pulpa o predadores de semillas, si picotean los frutos para consumir trozos de pulpa o depredar sus semillas. Estos procesos tienen consecuencias opuestas para el éxito reproductivo de las plantas. Por lo tanto, equiparar visitas a eventos de dispersión de semillas podría llevar a inferencias sesgadas sobre la ecología, evolución y conservación de este mutualismo planta‐animal.
3. En este estudio usamos información de la historia natural sobre las consecuencias funcionales de interacciones pareadas planta‐ave para examinar los cambios en la estructura de siete redes europeas de frugivoría tras eliminar las visitas clasificables como interacciones no mutualistas (picoteo de pulpa y depredación de semillas). De acuerdo con el conocimiento existente sobre las estructuras contrastadas de redes mutualistas y antagonistas, formulamos hipótesis sobre los cambios esperados al eliminar las interacciones no mutualistas, como un mayor anidamiento y una menor especialización.
4. Las interacciones no mutualistas, con picoteadores de pulpa y predadores de semillas, aparecieron en las siete redes, representando el 21‐48% de todas las interacciones y el 6‐24% de la frecuencia total de interacción. Cuando se eliminaron estas interacciones, hubo aumentos significativos en algunas métricas a nivel de red, como la conectancia y el anidamiento, pero disminuciones en otras como la robustez. Estos cambios fueron generalmente pequeños, homogéneos entre redes y generados por reducciones del tamaño de la red. Por el contrario, los cambios en las métricas a nivel de especie fueron más variables y a veces grandes, con disminuciones significativas en el grado de interacción de las plantas, así como en su frecuencia de interacción, especialización y resiliencia a las extinciones de animales, mientras que la fuerza de interacción de las aves aumentó significativamente.
5. Los datos de visitas pueden sobreestimar la frecuencia real de los servicios de dispersión de semillas en las redes de frugivoría. Aquí mostramos cómo la incorporación de información de la historia natural sobre las funciones de las interacciones entre especies puede mejorar nuestra comprensión de los procesos que operan en las comunidades ecológicas. Nuestro enfoque categórico sienta las bases para un trabajo futuro que cuantifique las consecuencias funcionales de las interacciones a lo largo del continuo mutualismo‐antagonismo que se ha documentado para otras faunas frugívoras.

## 1 INTRODUCTION

Interspecific interactions play a crucial role in the ecological and evolutionary dynamics of populations and communities (Roemer, Donlan, & Courchamp, 2002; Thompson, 2009), determining energy fluxes and mediating key ecological functions, such as mycorrhizal‐mediated mineral nutrition and animal‐mediated pollination and seed dispersal (Bascompte & Jordano, 2013). During the last decade, networks have increasingly been used to study the complex web of interactions that structure ecological communities (Heleno et al., 2014). The network approach allows ecologists to simultaneously “see the forest and the trees” (Heleno et al., 2014), that is, to analyse emergent properties at the community level while also assessing the functional role of individual species within communities. For example, the analysis of network‐level metrics has shown that mutualistic networks are more nested than antagonistic networks (Thébault & Fontaine, 2010), that specialization of pollination and frugivory networks decreases with latitude (Schleuning et al., 2012) and that non‐native frugivores have more connected and generalized interactions with local fleshy‐fruited plant communities than native frugivores (García, Martínez, Stouffer, & Tylianakis, 2014). Species‐level metrics have revealed, for example, that the role of invasive species within plant–pollinator networks can be predicted by their role in networks from their native range (Emer, Memmott, Vaughan, Montoya, & Tylianakis, 2016) and that dependence of frugivore species on fruits is positively related to their strength in seed dispersal networks (Fricke, Tewksbury, Wandrag, & Rogers, 2017).

There is, however, growing interest in understanding the functional role of species interactions in ecological networks (Ballantyne, Baldock, & Willmer, 2015; Heleno et al., 2014; King, Ballantyne, & Willmer, 2013). Yet, many networks are sampled by direct observation (Jordano, 2016). For example, pollination and seed dispersal networks are generally sampled by observing animals visiting plants to feed on their flowers or fruits (Chacoff et al., 2012; Plein et al., 2013). In both these mutualisms, visits describe food intake in animals, but not necessarily pollination or seed dispersal in plants. This issue has recently been examined for plant–pollinator interactions, showing that visitation does not necessarily mean effective pollination (Ballantyne et al., 2015; King et al., 2013). Consequently, network structure can change when incorporating detailed information on the functional outcomes of species interactions (Ballantyne et al., 2015; Carlo & Yang, 2011).

**Figure 1:**
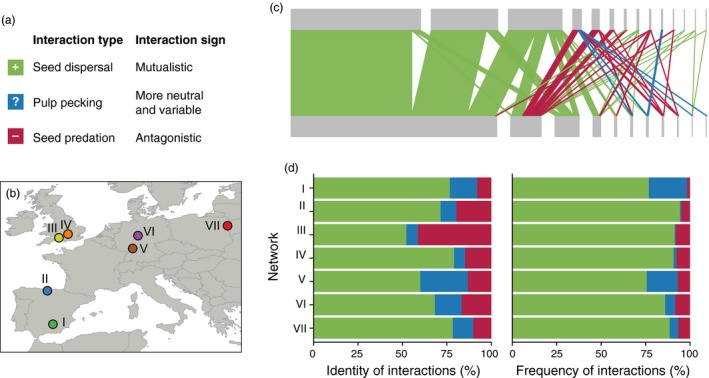
(a) Types of interactions between avian frugivores and fleshy fruits, and sign of the interaction from the plant's perspective. (b) Location and codes (roman numbers and colours) of the bird‐fruit visitation networks included in this study (I: P. Jordano, *unpublished*; II: García, 2016; III: Sorensen, 1981; IV: Snow & Snow, 1988; V: Plein et al., 2013; VI: Stiebel & Bairlein, 2008; VII: Farwig et al., 2017). (c) Representation of one of the studied networks (III); note that some frugivore species can have different interaction types depending on the plant species they feed on. (d) Frequency (%) of the different interaction types in the studied networks in terms of identity and quantity [Colour figure can be viewed at http://wileyonlinelibrary.com]

To our knowledge, no study has evaluated this issue in plant–frugivore networks (but see Genrich, Mello, Silveira, Bronstein, & Paglia, 2017; Montesinos‐Navarro, Hiraldo, Tella, & Blanco, 2017), despite research suggesting that it could be important (Albrecht, Neuschulz, & Farwig, 2012; Farwig, Schabo, & Albrecht, 2017; González‐Varo, 2010; Jordano, 1994; Jordano & Schupp, 2000; Snow & Snow, 1988). For plants, fleshy fruits represent the reward they offer for effective seed dispersal by animals (endozoochory), while for animals, fruits and seeds represent sources of food (Herrera, 2002; Janzen, 1983; Jordano, 2013). Frugivorous animals, notably birds and mammals, can process fleshy fruits by either (1) swallowing entire fruits and defecating or regurgitating viable seeds (legitimate seed dispersers) or (2) pecking or biting fruits for their pulp (pulp peckers) or seeds (seed predators) (see Snow and Snow 1988). Legitimate seed dispersers are true mutualists as they disperse plant progenies away from the maternal environment and allow the colonization of new sites (Traveset, Heleno, & Nogales, 2014). Conversely, seed predators are antagonists that destroy plant progeny (up to *c*. 80% in some plant populations; González‐Varo, 2010). Pulp peckers are between these two extremes (Figure 1a) because they neither disperse nor destroy seeds; they usually peck fruit, and the seed eventually drops to the ground (Jordano & Schupp, 2000). Some frugivore species may exhibit combinations of these behaviours when feeding on specific fruit species, falling into a continuum of interaction outcomes (Perea, Delibes, Polko, Suárez‐Esteban, & Fedriani, 2013). Clearly, frugivore visitation and seed dispersal are not equivalent, and plant reproductive success can be strongly influenced by the relative frequency of each type of interaction with frugivores (Schupp, Jordano, & Gomez, 2010). We may envisage a gradient of outcomes, depending on the particular pairwise interaction; the above categories representing a categorical summary of variable, context‐dependent outcomes.

Importantly, most plant–frugivore networks analysed in recent studies, and those available in open‐access network repositories, such as the Web of Life (http://www.web-of-life.es), are visitation networks (e.g. 16 of 18 in Schleuning et al., 2014), which include both pulp‐pecking and seed predation interactions (see Figure 1). This may not be a problem for questions related to the trophic specialization of frugivores (Dalsgaard et al., 2017). However, many studies using these networks aim to understand seed dispersal at the community level (Pigot et al., 2016; Schleuning et al., 2012, 2014) and its resilience to global change pressures (Fortuna & Bascompte, 2006; Schleuning et al., 2016), as well as identifying frugivore species that contribute the core of seed dispersal services (Fricke et al., 2017). Therefore, assessing structural differences between plant–frugivore visitation networks and true seed dispersal networks is important because strong biases might lead to incorrect inferences about the ecology, evolution and conservation of this mutualism.

Here, we classify all pairwise “bird–fruit” interactions in seven European “bird–fruit” visitation networks, as seed dispersal, pulp pecking or seed predation. We then evaluate how network structure and species structural roles (see Table 1) changed after removing the non‐mutualistic interactions (seed predation and pulp pecking). We focus on European networks because they share a biogeographical region (Western Palearctic; Figure 1b) and there is detailed natural history information available on the functional outcome of each pairwise bird–fruit interaction (e.g. Snow & Snow, 1988). Such information is crucial because the functional role of some bird species can change depending on the fruit species they feed on. It is important to note that our approach is primarily focussed on the fruit removal stage (the “departure stage”) of plant–frugivore interactions (Herrera, 2002), an easily obtainable proxy for actual seed dispersal success. However, true dispersal not only requires viable seeds to be removed from a plant, but also for seeds to be dispersed to suitable locations. Therefore, a complete assessment of seed dispersal effectiveness requires consideration of post‐removal processes, from seed deposition to seedling establishment (Schupp, Jordano, & Gómez, 2017; Spiegel & Nathan, 2010; Wenny & Levey, 1998).

**Table 1:** Network‐ and species‐level metrics considered in this study

Metrics (level)	Definition	Hypothesized change after removal of non‐mutualistic interactions
NETWORK LEVEL		
Size	The total number of species in the network	Decrease: due to the removal of exclusively non‐mutualistic frugivore species and plant species that only interacted with non‐mutualistic frugivores
Weighted connectance	Linkage density divided by the total number of species in the network (Tylianakis, Tscharntke, & Lewis, 2007)	Increase: due to (i) decrease in network size and (ii) because antagonists are expected to have a narrower niche than mutualists, and therefore, lower degree, suggesting that their removal should result in connectance increase (Blüthgen et al., 2007)
Weighted nestedness	Weighted NODF: a quantitative index for nestedness. Higher values indicate greater nestedness (Almeida‐Neto & Ulrich, 2011)	Increase: mutualistic systems tend to be nested, while antagonistic systems tend to be modular (Fontaine et al., 2011; Thébault & Fontaine, 2010). Therefore, when removing antagonistic interactions, we expect an increase in nestedness
*H*_2_′	A measure of network specialization. It ranges between 0 (no specialization) and 1 (complete specialization)	Decrease: predators tend to be more specialized than mutualists; therefore, specialization decreases when they are removed (Fontaine et al., 2011; Morris et al., 2014)
Weighted modularity	The LPAwb+ algorithm, a measure of community partitioning in quantitative networks (Beckett, 2016)	Decrease: antagonistic systems tend to be more modular than mutualistic systems (Fontaine et al., 2011; Thébault & Fontaine, 2010). Therefore, when removing antagonistic interactions, we expect a decrease in modularity
Robustness	Area under the curve of bird species removed vs. plant species remaining	Decrease: with fewer animal partners, on average plants will have less redundancy and undergo dispersal failure sooner. Therefore, robustness will be lower
SPECIES LEVEL (PLANTS)		
Degree	The number of species a given plant species interacts with	Decrease: any plant species with non‐mutualistic partners will undergo a decrease in degree. Plant species which exclusively interact with mutualistic partners will undergo no change in degree. Therefore, on average, a decrease is expected
Interaction frequency	The total interaction frequency of a given species	Decrease: any plant species with non‐mutualistic partners will undergo a decrease in interaction frequency due to a decrease in degree. Plant species which exclusively interact with mutualistic partners will undergo no change in interaction frequency. Therefore, on average, a decrease is expected
*d′*	Specialization of a species, measured as deviation from a random selection of its partners (Blüthgen et al., 2006)	Decrease: predators tend to be more specialized than mutualists; therefore, specialization decreases when they are removed (Blüthgen et al., 2007)
Resilience (*R*_75_)	The number of animal partners that are lost before a given plant species undergoes dispersal failure	Decrease: decreases in degree and interaction frequency mean that fewer partners will need to be removed until a plant species undergoes dispersal failure, resulting in a resilience decrease
SPECIES LEVEL (FRUGIVORES)		
Species strength	Sum of dependencies of plant species (Bascompte et al., 2006). It quantifies a frugivore species’ relevance across all the fleshy‐fruited plant community	Increase: plants will depend more on seed dispersers because dependencies in the original networks are distributed among mutualists and non‐mutualists; after the removal of non‐mutualistic interactions, dependencies will be spread among fewer partners and will, therefore, on average, be higher

## 2 MATERIALS AND METHODS

### 2.1 Study networks

We assembled a database of 1,051 plant–frugivore interactions from seven European quantitative visitation networks (Figure 1b). Some interactions occurred in more than one network, resulting in 681 unique interactions between 62 bird species spanning 19 families and 69 plant species from 23 families. All interactions were between plants and birds, except four plant–mammal interactions in network VII, which were excluded from subsequent analyses.

In five networks (I–V), interaction weights were visitation frequency. In the other two networks (VI and VII), weights were visitation rates, which were converted to visitation frequency by multiplying the rate for a plant species by time spent sampling that species. Where this did not result in an integer, values were rounded to the nearest whole number. We used visits as interaction weights because (1) it allowed use of quantitative null models, which require integer data, and (2) weights had to be standardized across all seven networks, and visitation rates were not available for networks I–V.

### 2.2 Interaction classification

We classified each bird–plant interaction as “seed dispersal,” “pulp pecking” or “seed predation”. Generally, a given bird species fits into one of these categories (Herrera, 1984). However, we did the classification at the interaction level because a bird species can have different interaction types depending on the plant species it feeds on (Figure 1c; Snow & Snow, 1988). For example, the Woodpigeon (*Columba palumbus*) can disperse large seeds with a hard coat (González‐Varo, Carvalho, Arroyo, & Jordano, 2017), but its gut typically destroys smaller and weaker seeds (Snow & Snow, 1988). Some bird species can even have different interaction types with the same plant species (Jordano & Schupp, 2000; Snow & Snow, 1988), but detailed data at the fruit level from network VII allowed us to validate our three‐category classification according to the predominant interaction type (Figure S1).

Classification data were directly available for 498 unique interactions (73.1%). Data came from four of the seven studied networks, namely *Birds and Berries* (Snow & Snow, 1988) for network IV and unpublished information from networks I (P. Jordano, *unpublished*), II (García, 2016) and VII (Farwig et al., 2017). For the remaining 183 unique interactions (26.9%), we inferred the interaction type from the above sources and other references (Simmons et al., 2018). Inference was based on interactions with congeneric species and/or interactions with plant species with fruits and seeds of similar size and type (such as drupe or berry). For example, we inferred that the Greenfinch (*Carduelis chloris*) consumed *Sorbus aria* seeds because one data source (Snow & Snow, 1988) classified greenfinches as predators of similar *Sorbus aucuparia* seeds.

### 2.3 Network‐level analysis

We first assessed how the removal of non‐mutualistic interactions changed network structure at the whole‐network level. We evaluated changes in six network‐level metrics commonly used in ecological studies (network size, weighted connectance, weighted nestedness, *H*_2_′, modularity and robustness) each of which we hypothesized to change in a certain direction following interaction removal (see Table 1 for metric definitions and their associated hypotheses). For each metric, we calculated its value (1) for the original visitation network with all interactions and (2) after the removal of the non‐mutualistic interactions (predatory and pulp‐pecking interactions). Many network metrics are sensitive to changes in network size. To control for this, we additionally used a null model approach, where metric values were Δ‐transformed. In Δ‐transformation, the mean value of a metric across 1,000 null networks is subtracted from the empirical network metric value, to describe the extent to which the metric deviates from a random expectation (Dalsgaard et al., 2017). We used two null models: the Patefield model, which constrains network size and marginal totals, and “quasiswap_count”, which constrains network size, marginal totals and connectance (the proportion of species pairs that interact in the network; Oksanen et al., 2016). The Patefield algorithm can generate unrealistic degree distributions and inflated Type II error rates (Bascompte & Jordano, 2013). However, the issue of null model building for networks is still unresolved, and currently, there is no better alternative than running different null models, some more conservative, others less conservative. That is the approach we have taken here: we use both the Patefield algorithm and the less conservative “quasiswap_count” algorithm.

We used one‐tailed Wilcoxon paired rank tests to determine whether network metrics consistently decreased or increased following the removal of non‐mutualistic interactions. We used one‐tailed tests because we adopted a hypothesis‐driven approach to test directional changes in network metrics. For example, we did not test whether nestedness changed in any direction after interaction removal; instead, we explicitly tested whether nestedness increased. This is because we hypothesized an increase in nestedness following the removal of non‐mutualistic interactions as mutualistic systems tend to be nested (Fontaine et al., 2011; Thébault & Fontaine, 2010). We adopted this approach for all metrics, with the hypothesized direction of change (and consequently the direction of the one‐tailed tests) given in Table 1. Additionally, we carried out one‐tailed Spearman's rank correlation tests to test whether the ranking of networks for each metric differed following interaction removal. A positive Spearman's correlation between metric values before and after removal is expected if there are no changes in ranks (assemblages respond to the removal of non‐mutualistic interactions in a consistent way), whereas such correlation is not expected if there are significant changes in ranks. Therefore, the direction of the tests was informed by the null hypothesis of no change in the ranks (an expected positive relationship). We consider a non‐significant Spearman's ρ to indicate a change in the ranks across networks. All these analyses were performed for the absolute metric values and the two sets of null‐corrected values.

To understand the processes driving changes in network metrics, we again used one‐tailed Spearman's rank correlation to test whether the magnitude of the change in network metrics following interaction removal correlated with the proportion of non‐mutualistic links removed from the networks. The direction of the one‐tailed test is determined by the hypotheses in Table 1.

As we conduct multiple tests, there is an increased probability of incorrectly rejecting the null hypothesis of no change in network metrics (Type I errors). We used the equation given by Moran (2003), based on a Bernoulli process, to calculate the probability of a given number of significant tests from a given number of trials. The probability, *p*, is given by the equation

$$p\;=\;\left[N!/\left(N\;-\;K\right)!K!\right]\;\times \;\alpha^{K}{\left(1\;-\;\alpha \right)}^{N-K},$$

where *N* is the number of tests conducted, and *K* is the number of tests below the significance level α.

### 2.4 Species‐level analysis

We assessed how the removal of non‐mutualistic interactions affected individual species, by examining changes in five species‐level metrics: four involving plant species (degree, interaction frequency, *d′* and resilience) and one involving frugivore species (species strength) (see Table 1 for metric definitions and their associated hypotheses). We calculated metric values for all species in all networks (1) in the original visitation networks with all interactions and (2) after the removal of all non‐mutualistic interactions. If interaction removal caused a species to lose all its links, it has a degree of zero and an interaction frequency of zero. We retained metric values of degree and interaction frequency for species that lost all links as excluding these would lead to an underestimation of mean changes in both metrics. However, the other metrics used in our analyses have a value of NA for a species with no links. We therefore excluded these NA metric values. We used one‐tailed Wilcoxon signed rank tests to determine whether metrics changed significantly following interaction removal. We performed tests for all species pooled together and separately for each network. The direction of the tests was informed by the hypothesized direction of change in each metric, as stated in Table 1. We additionally tested whether the ranking of species for each metric differed following interaction removal using one‐tailed Spearman's rank correlation tests. The direction of the tests was informed by the null hypothesis of no change in the ranks, therefore, an expected positive relationship. We consider a non‐significant Spearman's ρ to indicate a change in the ranks across networks.

### 2.5 Metric calculation

All network metrics and null models, except modularity, robustness and Resilience_75_, were calculated using the R package “bipartite” version 2.06.1 (Dormann, Fründ, Blüthgen, & Gruber, 2009; R Core Team, 2015). Modularity was calculated using the LPAwb+ code available on GitHub (https://github.com/sjbeckett/weighted-modularity-LPAwbPLUS; Beckett, 2016). For each modularity calculation, the LPAwb+ algorithm was run once. However, due to the stochastic nature of the algorithm, we also repeated our analyses, running the LPAwb+ algorithm 1,000 times for each modularity calculation. All results were unchanged by this additional analysis. Robustness and Resilience_75_ were calculated using a topological coextinction model, similar to that developed by Schleuning et al. (2016). In this model, we removed bird species in order of least to most interaction frequency (a proxy for abundance), as low abundance species are likely to be most vulnerable to anthropogenic pressures (Pimm, Lee Jones, & Diamond, 1988). Plant species were considered to have undergone dispersal failure when they had lost 75% of their interaction frequency. Robustness was measured as the area under the curve of bird species removed vs. plant species remaining, producing a value between 0 and 1 (Burgos et al., 2007; Pocock, Evans, & Memmott, 2012). Resilience of a given plant species was measured as the proportion of bird species that had to be removed from the network for it to undergo dispersal failure (Resilience_75_).

### 2.6 Removing only truly antagonistic (seed predation) interactions

We also performed all the analyses described above when only removing predatory interactions from the original visitation networks (leaving pulp‐pecking and seed dispersal interactions). This was because several of our hypotheses consider true antagonism (Table 1), whereas pulp peckers can be considered cheaters rather than antagonists because they do not destroy seeds and may exceptionally disperse seeds (Jordano & Schupp, 2000; Snow & Snow, 1988). This could affect changes in network metrics as the extent of modularity and nestedness in antagonistic networks is closely related to the degree of interaction intimacy (Pires & Guimarães, 2013). This is very generalized for pulp peckers (insectivores) but specialized for seed predators (granivores), like finches, whose bill morphology determines the size of seeds they can break and eat (Newton, 1967).

## 3 RESULTS

### 3.1 Prevalence of non‐mutualistic interactions

We found that both predatory and pulp‐pecking interactions occurred in all seven communities, although their prevalence varied among networks (Figure 1). Non‐mutualistic interactions comprised between 21% and 48% of links and between 5.7% and 24% of interaction frequency (Figure 1). Predatory interactions comprised between 8% and 41% of links and between 1.6% and 8.3% of interaction frequency. Pulp‐pecking interactions comprised between 6.2% and 26% of links and between 0.6% and 22% of interaction frequency.

**Figure 2:**
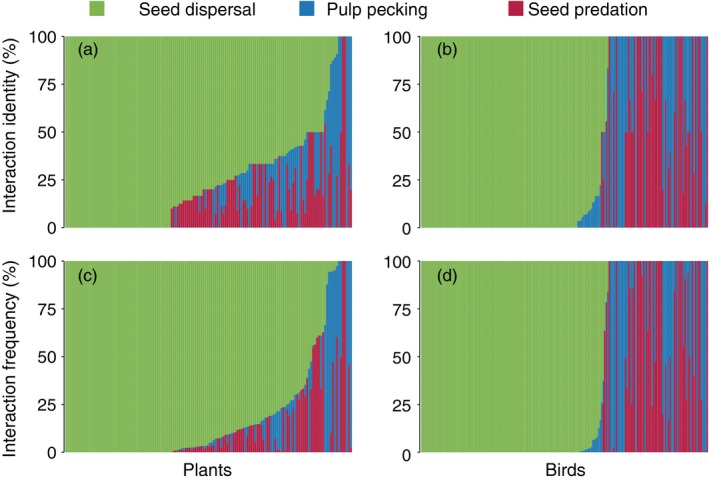
The composition of species’ links (a, b) and interaction frequency (c, d) for each plant (a, c) and bird (b, d) species across all networks. Each bar shows the proportion of a species’ links or interaction frequency which are seed dispersal (mutualistic), pulp pecking or seed predation (non‐mutualistic). Species are placed in order of decreasing proportion of links or interaction frequency, which are seed dispersal [Colour figure can be viewed at http://wileyonlinelibrary.com]

At the species level, we found that 63.2% of plant species were involved in non‐mutualistic interactions (between 48.0% and 90.9% of species in each network; Figure 2a,c). For birds, we found that 45.6% of species were involved in non‐mutualistic interactions (between 26.7% and 62.1% of species in each network; Figure 2b,d). The proportion of species’ links and interaction frequency that was seed dispersal, pulp pecking and seed predation is shown in Figure 2; the distribution of non‐mutualistic interactions is negatively skewed, but for many species constitutes a meaningful proportion. This is particularly true for bird species where 34.7% of species have no seed dispersal interactions.

Almost 80% of the interaction frequency with seed dispersers involved just two bird families (Turdidae: 66.9%; Sylviidae: 12.3%). Two bird families also accounted for 77%–78% of the interaction frequency with pulp peckers (Paridae: 49.2%; Fringillidae: 28.5%) and seed predators (Paridae: 27.1%; Fringillidae: 50.0%).

### 3.2 Changes in network‐level metrics

**Figure 3:**
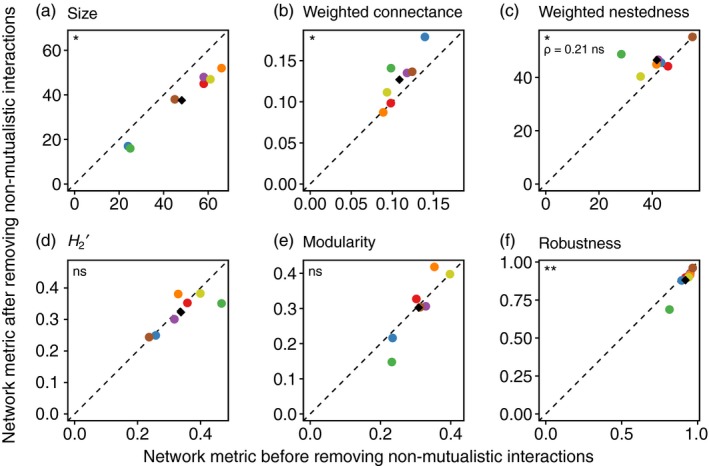
Changes (*y*‐axes) in the studied network‐level metrics after the removal of non‐mutualistic interactions (seed predation and pulp pecking). Colour codes denote network identity (see Figure 1b). The black diamonds are mean values across networks. The dashed line is *y *= *x*, indicating the position of points if there was no change in metric values. The significance of Wilcoxon matched pairs tests is shown in the top‐left corner of the panels (*ns*: non‐significant; *p < .05; **p <* *.01). Unless specified, all Spearman's ρ are significant (ρ ≥ .75, *p *<* *.05); we consider a non‐significant ρ to indicate a change in the ranks across networks [Colour figure can be viewed at http://wileyonlinelibrary.com]

We found small, but consistent, changes in four network‐level metrics after removing non‐mutualistic interactions (Figure 3; Table 2). Network size (Figure 3a) and robustness (Figure 3f) decreased significantly when interactions were removed, while weighted connectance (Figure 3b) and weighted nestedness (Figure 3c) increased significantly. No significant changes were found in *H*_2_′ (Figure 3d) or modularity (Figure 3e). The probability of finding four significant changes from six trials at a .05 significance level is .0000846 (Moran, 2003). Therefore, despite the inflated Type I error rate resulting from multiple tests, the number of significant results we found was substantially greater than expected from chance alone. In addition, we found a non‐significant rank correlation between the original and the modified network for weighted nestedness, indicating that removal of non‐mutualistic interactions led to changes in ranks across networks (Figure 3c).

**Table 2:** Mean change and variation in raw network‐level metrics following the removal of non‐mutualistic interactions

Metric	Mean (absolute)	Mean (%)	Range across networks	Coefficient of variation (%)
Size	−**10.57**	−**23.5**	−14 to −7	29
Weighted connectance	**0.02**	**16.2**	0.00 to 0.04	95
Weighted nestedness	**4.77**	**15.0**	−1.72 to 20.33	152
*H*_2_′	−0.01	−2.9	−0.12 to 0.05	336
Modularity	−0.01	−4.0	−0.08 to 0.06	698
Robustness	−**0.04**	−**4.5**	−0.13 to −0.01	103

Significant changes are shown in bold (.05 significance level).

When null models were used to control for changes in network size, changes in weighted connectance and weighted nestedness were not significant (Figure S4). This indicates that the significant changes in these metrics were driven by the decrease in network size. Conversely, decreases in robustness were still significant when corrected using both null models (Figure S4). This indicates that changes in robustness were more than expected from the size decrease alone and were driven by structural changes beyond those in connectance, as the “quasiswap_count” null model algorithm constrains size, marginal totals and connectance.

In general, the magnitude of the changes was not significantly related to the proportion of links removed (Figure S2). The exception was robustness, which significantly decreased with proportion of links removed (Spearman's ρ = −.71, *p *=* *.044; Figure S2).

### 3.3 Changes in species‐level metrics

**Figure 4:**
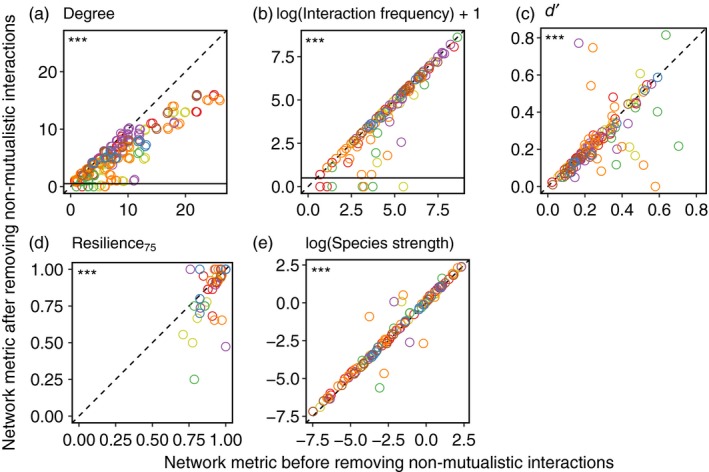
Changes (*y*‐axes) in species‐level metrics for plants (a–d) and frugivores (e) after the removal of non‐mutualistic interactions (seed predation and pulp pecking). Colour codes denote network identity (see Figure 1b). The dashed line is *y *= *x*, indicating the position of points if there was no change in metric values. Points below the horizontal black lines in panels (a) and (b) highlight those species that lose all their partners (a: degree) and interactions (b: frequency) after pruning. The significance of Wilcoxon matched pairs tests is shown in the top‐left corner of the panels (***p <* *.001)

At the species level, we found that several metrics significantly changed following the removal of non‐mutualistic interactions (Figure 4) and that these results were generally consistent across networks (see Tables 3 and 4). On average, species lost 2.1 partners (26.4%). Remarkably, the maximum change in plant species degree was −11. Additionally, some plant species lost all their links: this phenomenon occurred in four networks, affecting between 3.3% and 27.0% of plant species. There were significant decreases in plant degree, interaction frequency, *d′* and Resilience_75_ (Figure 4a–d), while frugivore species strength significantly increased (Figure 4f). Results for each network separately largely agree with the overall Wilcoxon results (Table 4), although a few metrics in some networks were unaffected by removal of non‐mutualistic interactions (Table 4). Finally, in one network, one metric differed in its rank following interaction removal: the Spearman's rank test for *d′* in network III was not significant (ρ = .50, *p *=* *.108), indicating that species’ relative values of *d′* changed following removal of non‐mutualistic interactions.

**Table 3:** Changes and variation in species‐level metrics following the removal of non‐mutualistic interactions

Metric	Mean (absolute)	Mean (%)	Range across networks (species)	Coefficient of variation (%)
Degree (plants)	−**2.10**	−**26.4**	−3.26 to −1.40 (−11 to 0)	121 (87–177)
Interaction frequency (plants)	−**44.26**	−**19.8**	−69.34 to −7.86 (−1,373 to 0)	294 (78–423)
*d′* (plants)	−**0.03**	−**11.4**	−0.12 to −0.01 (−0.58 to 0.60)	464 (80–1,254)
Resilience_75_ (plants)	−**0.03**	−**3.6**	−0.13 to 0.00 (−0.54 to 0.24)	346 (138–2,103)
Species strength (frugivores)	**0.14**	**35.40**	0.04 to 0.33 (−0.76 to 2.19)	282 (143–305)

Significant changes are shown in bold (.05 significance level). The mean change in each metric for each network was calculated, and then, an overall mean was obtained by calculating the mean of the mean changes in each network. The range of the mean change across networks is also reported, as well as the range of change across species in parentheses. The coefficient of variation was calculated across all species in all networks; in parentheses, we show the range of coefficients of variation when calculated for each network separately.

**Table 4:** Results of species‐level Wilcoxon tests per network (I–VII) for each metric

Metric	Change	I	II	III	IV	V	VI	VII
Degree (plants)	−	**	*	**	***	***	***	**
Interaction frequency (plants)	−	**	*	**	***	***	***	**
*d′* (plants)	−	*	**	*	***	ns	***	***
Resilience_75_ (plants)	−	**	ns	*	ns	*	**	ns
Species strength (frugivores)	+	***	**	*	**	**	***	***

“+” indicates that the metric increased following the removal of non‐mutualistic interactions, while “−” indicates a decrease.

*, ** and *** denote *p *<* *.05, *p *<* *.01 and *p *<* *.001, respectively (ns: non‐significant differences).

### 3.4 Removal of seed predation interactions

When only seed predator interactions were removed, changes in network‐ and species‐level metrics followed the same direction as when all non‐mutualistic interactions were removed, although they were smaller in magnitude (see Appendix S1). Results of “quasiswap_count” null models showed that changes in *H*_2_′ and weighted nestedness were driven by changes in network structure rather than just decreases in network size (Appendix S1). Moreover, changes in *H*_2_′ (ρ = −.86, *p *=* *.012) and modularity (ρ = −.79, *p *=* *.024) were significantly negatively correlated with the proportion of links removed from the original visitation networks (Appendix S1). Weighted connectance and weighted nestedness were positively related to the proportion of links removed from the original networks, yet these correlations were marginally significant (ρ = .61 and *p *=* *.083 for both metrics; Appendix S1).

## 4 DISCUSSION

Here, we disentangled seed dispersal interactions (mutualism) from pulp‐pecking (exploitation) and seed predation (antagonism) interactions in European plant–frugivore networks and evaluated changes in network properties when removing non‐mutualistic interactions. We found that, at the network level, the magnitude of most changes was small and relatively uniform, suggesting that studies treating plant–frugivore visitation networks as seed dispersal networks are likely robust if they only use network‐level metrics (although consideration of processes acting after fruit removal is strictly necessary to infer true dispersal). However, for species‐level metrics, changes were generally larger and more variable, indicating the importance of considering natural history to gain insights into the functional roles of species in seed dispersal mutualisms. Ignoring such functional outcomes in visitation networks at the species level may, for instance, overestimate the potential for functional redundancy across frugivore species in the assemblage and the potential for interaction rewiring after loss of frugivore partners.

### 4.1 Changes in network‐level metrics

European seed predators and pulp peckers feed on fleshy fruits less frequently than legitimate seed dispersers, which likely explains why non‐mutualistic interactions were generally more important in qualitative than quantitative terms. Seed predators have bill morphologies poorly adapted for frugivory, which increases fruit‐handling times and lowers energy intake, while pulp peckers’ long gut passage time makes fruit an inefficient food source due to its low nutrient content per unit mass (Herrera, 1984). Instead, non‐disperser species primarily feed on seeds from dry fruits or insects (Herrera, 1984). This tendency for predatory and pulp‐pecking interactions to constitute a relatively small proportion of interaction frequency may explain why network‐level metrics generally undergo only small changes after removing non‐mutualistic interactions: we have used weighted versions of network‐level metrics, where weaker interactions exert less influence on metric values than stronger interactions. Our results therefore suggest that macroecological studies comparing weighted network‐level metrics between multiple plant–frugivore visitation networks (e.g. Schleuning et al., 2012) are likely to be robust to the presence of non‐mutualistic interactions, especially when comparing *H*_2_′ and modularity. For example, Dalsgaard et al. (2017) examined latitudinal patterns in network specialization (*H*_2_′), finding values ranging between 0.18 and 0.48. Such values are an order of magnitude greater than the mean change in *H*_2_′ we observed when removing non‐mutualistic interactions, and so any biases are unlikely to affect the general conclusions of such studies. Studies are likely robust to changes in weighted and unweighted connectance and nestedness too, given the small magnitude of the mean absolute changes we found in these values (Figure S3, Table S1).

Removal of non‐mutualistic interactions can lead to decreases in network size in two ways: (1) frugivore species can be lost if they only form non‐mutualistic interactions with plant species, and (2) plant species can be lost if, during the sampling period, they only interact with frugivores that destroy their seeds or peck their pulp. The loss of purely non‐mutualistic frugivores was the main driver of changes in network size, although plant loss did affect four of the seven networks. The loss of frugivores also helps to explain the decrease in robustness when non‐mutualistic interactions were removed: with fewer frugivore species, plants have fewer partners and less redundancy, meaning that the removal of a single bird species causes plants to lose a greater proportion of their interaction frequency than in the pre‐removal network. Our results suggest that studies that do not consider interaction types may overestimate robustness and the redundancy of seed dispersal mutualisms and that this overestimation increases with the proportion of non‐mutualistic interactions in a community (Figure S2). Therefore, inferences about the sensitivity of seed dispersal processes to species loss need to carefully account for the natural history of pairwise interactions.

When removing only predatory interactions, changes in weighted nestedness and *H*_2_′ were greater than expected from the decrease in network size alone and were likely related to the antagonistic nature of the removed interactions. For example, the decrease in *H*_2_′ may be explained by antagonists forming more specialized interactions than mutualists (Fontaine et al., 2011; Morris, Gripenberg, Lewis, & Roslin, 2014). This is expected for seed predators because bill size (depth) determines the size of seeds that predators can break and eat (Newton, 1967). Similarly, the increase in nestedness is supported by a number of previous studies that found nested architectures to be more common in mutualistic than antagonistic networks (Fontaine et al., 2011; Thébault & Fontaine, 2010), a pattern driven by multiple ecological and evolutionary processes (Bascompte, 2010; Vázquez, Chacoff, & Cagnolo, 2009). For example, nestedness has been shown to stabilize mutualistic communities, but has a negative effect on the stability of antagonistic systems (Okuyama & Holland, 2008; Thébault & Fontaine, 2010). Conversely, high connectance is associated with stability in mutualistic systems, while antagonistic communities favour less connected architectures (Thébault & Fontaine, 2010). Therefore, with fewer antagonistic interactions and species, connectance and nestedness increase and specialization decreases. This suggests that, even though non‐mutualistic interactions make up only a fraction of plant–frugivore networks, the structure of these networks seems to have an imprint of the antagonistic interactions. Finally, the change in relative weighted nestedness that followed interaction removal could be partially due to variations in the prevalence of non‐mutualistic interactions (see trend in Figure 3c) and suggests that comparisons between networks can be confounded by such changes.

### 4.2 Changes in species‐level metrics

Changes in species‐level metrics were most clear for plant degree and interaction frequency, with many species losing interaction partners (Figure 4). In some cases, the loss of degree was extreme and so, particularly for some species, incorporating information on the functional outcomes of interactions greatly changes inferences about their ecology and evolution. These results suggest that plant species have weaker dispersal interactions with fewer partners than previously recognized. Overall, these differences translated into a small but significant decrease in mean plant resilience to animal removal of −.03. This value indicates that, after interaction removal, the average percentage of animal species that had to be removed from the network for plant species to undergo dispersal failure decreased by 3%. However, this mean value masks some heterogeneity in species responses (Table 3). Resilience can increase despite plant species having fewer partners on average if the removal of interactions changed the animal removal sequence or if non‐mutualistic interactions constituted a large proportion of a species’ interaction frequency in the original networks. Therefore, we conclude that, while most estimates of resilience are relatively unchanged by incorporating natural history information, some plant species underwent more major changes, revealing them as more susceptible to global change pressures, including climate change (Schleuning et al., 2016) or disperser extinction (Rumeu et al., 2017).

While decreases in plant *d*′ may initially seem counterintuitive, *d′* is a measure of the extent to which a species deviates from randomly sampling all available partners and so does not necessarily correlate with measures of specificity, such as degree (Blüthgen, Menzel, & Blüthgen, 2006). Instead, with *d′*, species with one partner can be less specialized than species with two partners. For example, if a plant is only visited by one frugivore species, but this frugivore is highly dominant in the community, the plant would have a low *d*′ value. Conversely, if a plant is visited by two very rare frugivores, it would have a high *d*′ value. Antagonistic relationships, such as those between predators and prey or between hosts and parasites, tend to have higher levels of specialization than mutualistic systems because hosts and prey deploy defences, which constrain the available partners of their enemies (Blüthgen, Menzel, Hovestadt, Fiala, & Blüthgen, 2007; Jaenike, 1990).

Increases in frugivore species strength (the sum of dependencies of plant species on frugivores [Bascompte, Jordano, & Olesen, 2006]) occurred because, before non‐mutualistic interactions were removed, plants distributed their dependencies among all avian frugivores. However, once non‐mutualistic interactions were removed, plant dependencies shifted entirely to the seed dispersers, thereby increasing their strength values.

### 4.3 Generalizations and limitations

Our analyses represent an attempt to disentangle the variety of mutualistic and antagonistic processes present in plant–frugivore networks to focus on the seed dispersal of plant communities by legitimate seed dispersers. Most “bird–fruit” interactions involving European frugivorous birds can be easily classified as “seed dispersal,” “pulp pecking” and “seed predation” thanks to (1) the availability of necessary data and (2) the fact that most birds fall within one category (Herrera, 1984; Snow & Snow, 1988). Exceptionally, a few frugivore species may exhibit dual roles (such as the European nuthatch *Sitta europaea*; Jordano & Schupp, 2000; see also Figure S1); and some pulp peckers may pluck fruits and peck them in the branch of a nearby tree, dispersing the seed a few metres (e.g. Great tit *Parus major*; Jordano & Schupp, 2000; Snow & Snow, 1988). Additionally, certain frugivores that predominantly act as pulp peckers (e.g. Great tit) and seed predators (e.g. Chaffinch *Fringilla coelebs*) have been reported to disperse seeds of fleshy fruits internally, through endozoochory (Cruz, Ramos, da Silva, Tenreiro, & Heleno, 2013). However, evidence from the gut content of road‐killed (Debussche & Isenmann, 1989) and mist‐netted birds (Olesen et al., 2011), and more recently from DNA barcoding applied to dispersed seeds (González‐Varo, Arroyo, & Jordano, 2014), demonstrates that seed predators and pulp peckers are virtually absent from true seed dispersal networks. These results suggest that networks sampled using methods other than observations of visits, such as by identifying seeds and/or pulp remains recovered from faeces, are likely to be closer in structure to the “true” seed dispersal networks revealed by removing non‐mutualistic interactions than the raw visitation networks containing non‐mutualistic interactions. Thus, analysing non‐visitation networks, such as seed deposition networks, could be a useful way to circumvent some of the issues raised by this study, bringing us closer to a description of plant–seed disperser community structure (Wang & Smith, 2002).

While our dataset covers a large spatial extent in Europe, further research, with a larger database of networks covering other regions, would help assess whether our conclusions hold for other parts of the world. However, we are aware that such simplistic classification may not work in other frugivore groups that fall into a mutualism–antagonism continuum, such as tanagers in the neotropics (Moermond & Denslow, 1985), parrots (Montesinos‐Navarro et al., 2017) and ungulate mammals (Perea et al., 2013). Whenever dealing with visitation data, the challenge in these groups is to quantify the frequency of different interaction outcomes with multiple plant species in order to incorporate weights of seed dispersal effectiveness into the links of the networks (Schupp et al., 2017).

Finally, while here we have incorporated information on fruit removal, it is important to remember that there remain other natural history details not included in this study. For example, birds of different sizes remove different quantities of seeds in a given visit, and therefore, one visit of a small bird is not equivalent to one visit of a larger bird in terms of seed removal (Carlo & Yang, 2011). While visitation frequency is a main component of interaction outcome in generalized plant–frugivore networks, per‐visit effects may overcome differences in visitation and alter frugivore effectiveness in significant ways.

## 5 CONCLUSIONS

Ecological networks constitute a powerful tool to analyse complex interactions between multiple species. We show here that adding more natural history details on the nature of species interactions can bring us closer to understanding the ecological processes and functions they mediate; here, seed dispersal mediated by frugivorous animals. After removing non‐mutualistic interactions, changes in network‐level metrics were generally small (particularly for *H*_2_′, modularity and robustness) and consistent. Importantly, consistent changes at the network level still allow for valid comparisons among networks. However, at the species level, changes tended to be larger and more variable. This makes it harder to anticipate how individual species might respond if non‐mutualistic interactions were removed: while some species may be unaffected, others are highly affected. Importantly, our results show that plants have less frequent interactions with fewer frugivores than previously recognized and with more limited ecological redundancy. Consequently, we advise caution when using species‐level metrics on plant–frugivore visitation networks whenever seed dispersal is the studied ecological process.

## AUTHORS’ CONTRIBUTIONS

B.I.S. and J.P.G.‐V. conceived the study; J.A., N.F., D.G. and P.J. provided empirical network data; B.I.S. and J.P.G.‐V. conducted the statistical analyses and wrote the first manuscript draft; all authors discussed the results, contributed during manuscript writing and approved the final manuscript.

## Data Availability

Original data associated with this article (classification of interaction outcomes for the 681 unique “bird–fruit” interactions) are deposited in Dryad Digital Repository: https://doi.org/10.5061/dryad.r3d70m9 (Simmons et al., 2018).
