# Supplementary material for: Moving from frugivory to seed dispersal: Incorporating the functional outcomes of interactions in plant–frugivore networks
Source: J Anim Ecol. 2018 Apr 20;87(4):995–1007. doi: 10.1111/1365-2656.12831 (PMC6849527; doi:10.1111/1365-2656.12831)
Supplement: Supplementary file 1 [file JANE-87-995-s001.docx]

Supplementary information

**Moving from frugivory to seed dispersal: incorporating the functional outcomes of interactions in plant-frugivore networks**

Benno I. Simmons^1*^, William J. Sutherland^1^, Lynn V. Dicks^1,2^, Jörg Albrecht^3^, Nina Farwig^4^, Daniel García^5^, Pedro Jordano^6^ and Juan P. González-Varo^1^

*^1^ Conservation Science Group, Department of Zoology, University of Cambridge, The David Attenborough Building, Pembroke Street, Cambridge, CB2 3QZ, UK*

*^2^ Biological Sciences, University of East Anglia, NR4 7TL, UK*

*^3^ Senckenberg Biodiversity and Climate Research Centre (BiK-F), Senckenberganlage 25, 60325 Frankfurt am Main, Germany*

*^4^ Conservation Ecology, Faculty of Biology, Philipps-University Marburg, Karl-von-Frisch Str. 8, 35043 Marburg, Germany*

*^5^ Dpto. de Biología de Organismos y Sistemas, Unidad Mixta de Investigación en Biodiversidad (CSIC-UO-PA), Universidad de Oviedo, C/ Catedrático Rodrigo Uría s/n, Oviedo, E-33006, Spain*

*^6^ Integrative Ecology Group, Estación Biológica de Doñana (EBD-CSIC), Avda. Américo Vespucio 26, Sevilla, E-41092, Spain*

***** Corresponding author: benno.simmons@gmail.com


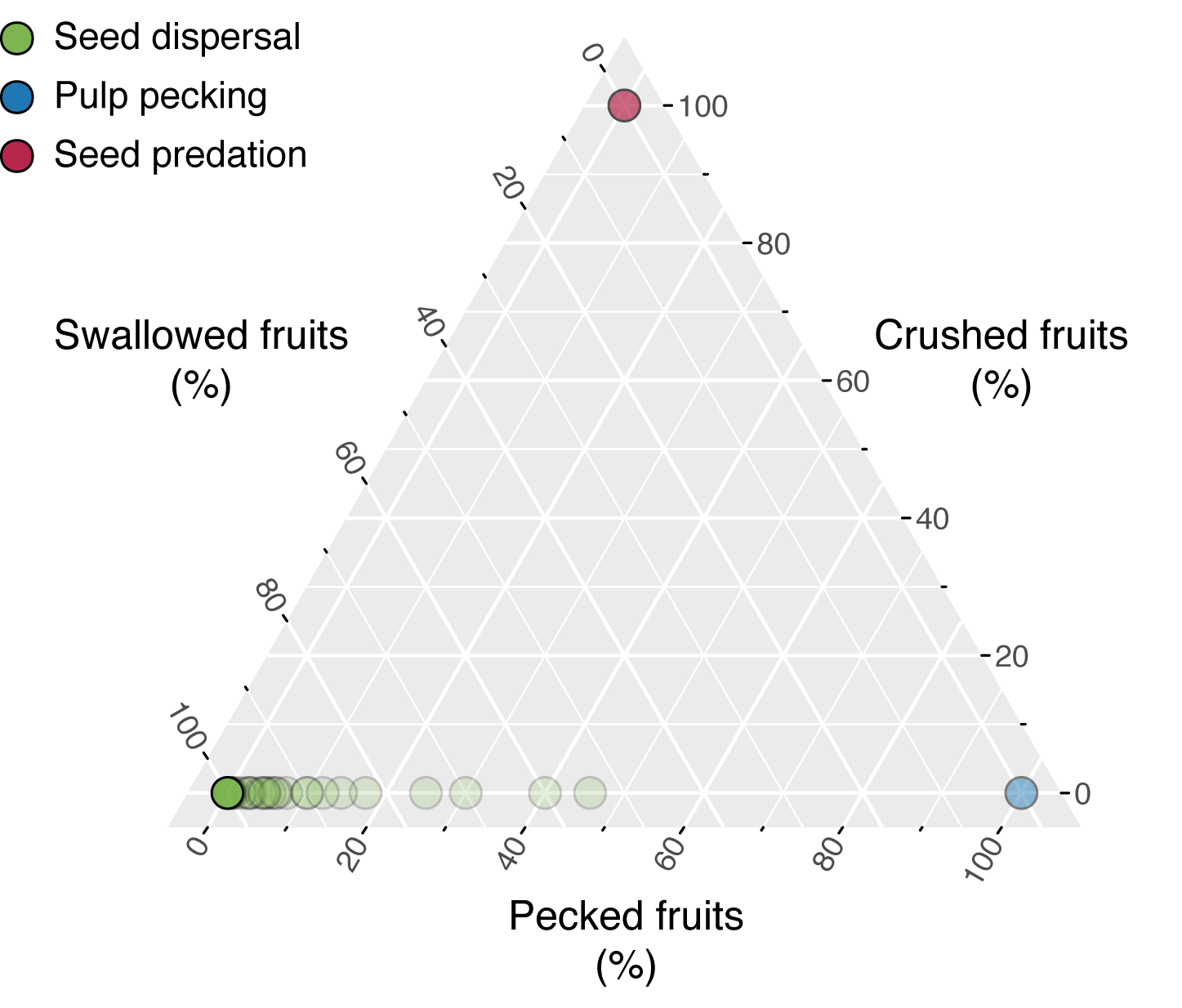


**Figure S1.** Ternary plot showing the percentage of fruits that were swallowed, pecked or crushed in 77 pairwise fruit-bird interactions from network VII (Farwig *et al*. 2017) that were classified as ‘seed dispersal’ (*n* = 69), ‘pulp pecking’ (*n* = 3) or ‘seed predation’ (*n* = 5). These 77 interactions – out of a total of 129 – were those for which the number of fruits observed to obtain the percentages was ≥ 6 (range = 6–1794 fruits, median = 46, total = 10,997). The figure illustrates that our categorical classification is, in general, very consistent with detailed information on the specific role of each pairwise interaction. In particular, when the interactions were classified as ‘seed predation’ there was no ‘seed dispersal’ or ‘pulp pecking’. Moreover, interactions classified as ‘seed dispersal’ only included small fractions of ‘pulp pecking’ (median = 0%, mean = 3.7%), and only in few cases this fraction was higher than 20% (max. = 45.7%). Plant-frugivore interactions were observed using binoculars from camouflage tents. During the observations all frugivore species visiting the individual plants were recorded, as well as the number of frugivore individuals, the duration of frugivore visits and fruit-handling behaviour (interaction outcome). Swallowing, (seed) crushing and (pulp) pecking were distinguished. Thereby, single visitors could handle fruits in various ways, so that some fruits were swallowed, crushed or pecked during the same visit. If a group of conspecific frugivores visited a plant and individual behaviour could not be observed simultaneously, the most visible individual was focused on. If the behaviour of different species could not be observed simultaneously, the rarer species was focused on.

**Figure S2.** Relationship between the difference (∆) in the network-level metrics after the removal of non-mutualistic interactions (seed predation and pulp pecking), and the proportion of interactions removed in the original networks. For illustrative purposes, we show a regression line in significant Spearman’s rank correlations (*ns*: non-significant; * *P* < 0.05).

**Figure S3.** Changes in unweighted (not incorporating information on interaction frequency) connectance and nestedness after the removal of non-mutualistic interactions. Colour codes denote network identity (see Fig. 1b). The black diamonds are mean values across networks. The dashed line is *y* = *x*, indicating the position of points if there was no change in metric values. The significance of Wilcoxon matched-pairs tests is shown in the top-left corner of the panels (*ns*: non-significant; * *P* < 0.05).

**Figure S4** Changes in the null-corrected (Δ-transformed) network-level metrics after the removal of non-mutualistic interactions (seed predation and pulp pecking). Colour codes denote network identity (see Fig. 1b). The black diamonds are mean values across networks. The dashed line is *y* = *x*, indicating the position of points if there was no change in metric values. The significance of Wilcoxon matched-pairs tests is shown in the top-left corner of the panels (*ns*: non-significant; * *P* < 0.05).

**Table S1.** Mean change and variation in weighted and unweighted connectance and nestedness following the removal of non-mutualistic interactions. Significant changes are shown in bold.

| Metric | Type | Change in metric | Change in metric (%) | Range | Coefficient of variation (%) |
| --- | --- | --- | --- | --- | --- |
| Connectance | Unweighted | **0.05** | **15.6** | –0.01 to 0.1 | 86 |
|  | Weighted | **0.02** | **16.2** | 0.00 to 0.04 | 95 |
| Nestedness | Unweighted | 2.98 | 12.4 | –1.17 to 14.77 | 188 |
|  | Weighted | **4.77** | **15.0** | –1.72 to 20.33 | 152 |

Appendix S1: Removing seed predation interactions only

In this Appendix, we repeated our analyses from the main text, but removing only seed predation interactions (leaving pulp-pecking and seed dispersal interactions). At the network level, changes were similar to removing all non-mutualistic interactions, though the magnitude of changes was smaller (Fig A1.1; Table A1.1). Size and *H*_2_′ decreased significantly when predatory interactions were removed, while weighted connectance and weighted nestedness significantly increased (Table A1.1). Change in *H*_2_′ and modularity were significantly related to the proportion of seed predator interactions that were removed from a network (Fig. A1.2), which likely reflects the more modular and specialised architecture of antagonistic systems. Weighted connectance, weighted nestedness and *H*_2_′ all significantly decreased even when corrected using null models, indicating that changes in these metrics were not solely driven by network size (Fig A1.3). This again is likely due to the contrasting structure of mutualistic and antagonistic systems. At the species level, changes were in the same direction as when all non-mutualistic interactions were removed but of smaller magnitude (Fig. A1.4; Table A1.2). Plant degree, interaction frequency, d’ and Resilience_75_ all decreased significantly, while frugivore species strength significantly increased. Changes were generally consistent across networks, though some metrics did not change significantly in some networks, despite having significant changes overall across all networks (Table A1.3).

**Figure A1.1.** Changes in the studied network-level metrics after the removal of seed predator interactions. Colour codes denote network identity (see Fig. 1b). The black diamonds are mean values across networks. The dashed line is *y* = *x*, indicating the position of points if there was no change in metric values. The significance of Wilcoxon matched-pairs tests is shown in the top-left corner of the panels (*ns*: non-significant; * *P* < 0.05). Unless specified, all Spearman’s *ρ* are significant (*ρ* ≥ 0.89, *P* < 0.05); we consider a non-significant *ρ* to indicate a change in the ranks across networks.

**Figure A1.2.** Relationship between the difference (∆) in the network-level metrics after the removal of seed predator interactions, and the proportion of interactions removed in the original networks. For illustrative purposes, we show a regression line in significant Spearman’s rank correlations (*ns*: non-significant; * *P* < 0.05).

**Figure A1.3.** Changes in the null-corrected (Δ-transformed) network-level metrics after the removal of seed predator interactions. Colour codes denote network identity (see Fig. 1b). The black diamonds are mean values across networks. The dashed line is *y* = *x*, indicating the position of points if there was no change in metric values. The significance of Wilcoxon matched-pairs tests is shown in the top-left corner of the panels (*ns*: non-significant; * *P* < 0.05; ** *P* < 0.01).

**Figure A1.4.** Changes in network-level metrics (a-d) and frugivores (e) after the removal of seed predator interactions. Colour codes denote network identity (see Fig. 1b). The dashed line is *y* = *x*, indicating the position of points if there was no change in metric values. Points below the horizontal black lines in panels (a) and (b) highlight those species that lose all their partners (a: degree) and interactions (b: frequency) after pruning. The significance of Wilcoxon matched-pairs tests is shown in the top-left corner of the panels (*** *P* < 0.001).

**Table A1.1.** Mean change and variation in network-level metrics following the removal of non-mutualistic interactions. Significant changes are shown in bold.

| Metric | Change in  metric | Change in  metric (%) | Range | Coefficient of variation (%) |
| --- | --- | --- | --- | --- |
| Size | **–3.86** | **–9.4** | –6 to –2 | 46 |
| Weighted connectance | **0.01** | **5.8** | 0 to 0.02 | 115 |
| Weighted nestedness | **2.44** | **6.0** | –0.48 to 8.79 | 127 |
| *H*_2_′ | **–0.01** | **–3.7** | –0.06 to 0 | 152 |
| Modularity | –0.02 | –7.3 | –0.08 to 0.02 | 153 |
| Robustness | –0.02 | –2.6 | –0.15 to 0.02 | 276 |

**Table A1.2.** Changes and variation in species-level metrics following the removal of predatory interactions. The mean change in each metric for each network was calculated. An overall mean was obtained by calculating the mean of the mean changes in each network. The range of the mean change across networks is also reported as well as the range of change across species in parentheses. The coefficient of variation was calculated across all species in all networks; in parentheses we show the range of coefficients of variation when calculated for each network separately.

| Metric | Mean (absolute) | Range | Coefficient of variation (%) |
| --- | --- | --- | --- |
| Degree (plants) | –1.16 | –1.73 to –0.48 (–7 to 0) | 138 (89 to 154) |
| Interaction frequency (plants) | –25.38 | –57.38 to –4.60 (–476 to 0) | 288 (86 to 308) |
| *d′* (plants) | –0.02 | –0.04 to 0.01 (–0.40 to 0.34) | 421 (96 to 3092) |
| Resilience_75_ (plants) | –0.02 | –0.15 to 0.03 (–0.59 to 0.24) | 689 (131 to 14986) |
| Species strength (frugivores) | 0.02 | –0.07 to 0.09 (–2.46 to 1.31) | 1056 (307 to 3778) |

**Table A1.3.** Results of species-level Wilcoxon tests per network for each metric; ‘+’ indicates that the metric increased following the removal of predatory interactions, while ‘–’ indicates a decrease. *, ** and *** denote *P* < 0.05, *P* < 0.01 and *P* < 0.001, respectively (ns: non-significant differences).

| Metric | Change | I | II | III | IV | V | VI | VII |
| --- | --- | --- | --- | --- | --- | --- | --- | --- |
| Degree (plants) | – | ** | * | ** | *** | *** | *** | * |
| Interaction frequency (plants) | – | ** | * | ** | *** | *** | *** | * |
| *d′* (plants) | – | ns | ** | ns | *** | * | *** | *** |
| Resilience_75_ (plants) | – | * | ns | * | ns | ** | * | ns |
| Species strength (frugivores) | + | *** | ns | ns | * | ** | ns | *** |
